# The safety and efficacy of Houtou Jianweiling tablet in patients with chronic non-atrophic gastritis: a double-blind, non-inferiority, randomized controlled trial

**DOI:** 10.3389/fphar.2024.1293272

**Published:** 2024-02-19

**Authors:** Muhammad Raza Shah, Samreen Fatima, Sehrosh Naz Khan, Shafiullah, Zahid Azam, Hafeezullah Shaikh, Shahid Majid, He Chengdong, Zhou Daijun, Wei Wang

**Affiliations:** ^1^ Center for Bioequivalence Studies and Clinical Research, Dr. Panjwani Center for Molecular Medicine and Drug Research, International Center for Chemical and Biological Sciences, University of Karachi, Karachi, Pakistan; ^2^ National Institute of Liver & GI Diseases (NILGID), DOW University of Health Sciences, Karachi, Sindh, Pakistan; ^3^ The Indus Hospital Karachi, Karachi, Sindh, Pakistan; ^4^ Hunan Xinhui Pharmaceutical Co., Ltd., Changsha, Hunan, China; ^5^ TCM and Ethnomedicine Innovation & Development International Laboratory, School of Pharmacy, Hunan University of Chinese Medicine, Changsha, China

**Keywords:** traditional Chinese medicine (TCM), Houtou Jianweiling tablet (HTJWT), omeperazole, randomization, clinical trial

## Abstract

**Background:** Common symptoms of Chronic Non-atrophic Gastritis (CNAG) include nausea, stomach distension, and abdominal pain. The Houtou Jianweiling Tablet (HTJWT) is a chinese patent medicine (CN1368229A) and it has been used clinically for more than 20 years with proven clinical efficacy in treating CNAG, prompted us to establish the clinical efficacy and safety of HTJWT on patients with mild to moderate CNAG symptoms in Pakistani population.

**Methods:** This phase II, double-blind, randomized, parallel-controlled trial was conducted in a single center between November 2022 and February 2023 in Pakistan. In a ratio of 1:1, total 240 CNAG patients with erosion identified by pathological biopsy and gastroscopy were randomly assigned to control (Omeprazole) group (*n* = 120) and the treatment (HTJWT) group (*n* = 120). Patients in the treatment group received orally four HTJWT (0.38g/tablet), three times a day and one placebo of Omeprazole enteric-coated tablet prior to breakfast, daily. On the other hand, patients in the control group received one Omeprazole enteric-coated tablet (20 mg/tablet) prior to breakfast and four placebo of HTJWT, thrice a day. The patients consumed the investigated drugs (i.e., treatment and control) treatment regimen was followed for a duration of 28 days. The safety of the patients were evaluated through adverse events, serious adverse events and laboratory tests such as blood biochemistry, urine analysis, liver and renal function tests. Vital signs like; blood pressure, pulse rate, body temperature, respiratory rate for all the patients were recorded. The cardiac status of the patients were assessed through electrocardiogram (ECG). The primary efficacy indicators were the improvement rate of gastric distention and gastralgia as the main clinical symptoms. Secondary indicators were visual analogue score (VAS); improvement rate of secondary clinical symptoms and signs; improvement rate of total clinical signs and symptoms; the disappearance/remission rate of Gastric pain and, remission/disappearance time of gastric distension; and the negative conversion rate of *Helicobacter pylori* (*H. pylori*). The outcomes among each group were compared using the chi-square test.

**Results:** Patients in both groups had good drug compliance (80%–120%), and there was no statistically significant difference in the patients’ baseline characteristics. The clinical improvement rate was found to be 91.1% in the treatment group and 91.0% in the control group with negligible variation among the two groups (*p* = 0.9824; 95% confidence interval: -0.0781–0.0798). Similarly, hardly no difference was found in the negative conversion rate of *H. pylori* between the treatment group and the control group (i.e., 70.1% and 71.8% respectively, *p* = 0.8125). There were no significant differences in respiratory rate, vital signs, blood pressure, laboratory results for blood biochemistry, urine analysis, liver and renal function tests between the two groups. The ECG assessment carried out for the treatment and control group revealed no considerable difference. Margin variation in the disappearance time of gastric pain (*p* = 0.1860) and remission rate (*p* = 0.5784) between the two groups were observed. The control group exhibited a faster remission period for gastrointestinal discomfort indications as compared to treatment group (*p* = 0.0430). Only one patient in the control group experienced mild to moderate adverse events, namely,; epigastric pain and dyspepsia. The results were consistent with the intention-to-treat and per-protocol analysis that included patients who were 100% compliant to the assigned therapy.

**Conclusion:** The lower limit of confidence interval (CI, 95%) for the differences in the effective rate between the treatment and the control groups was found to be −0.0781 which is greater than −0.15, hence the treatment group is non-inferior to the control group. The therapeutic dosage used in the trial and treatment period did not cause any significant adverse event, and there were no obvious changes in the ECG profile, vital signs and biochemistry of the patients. Based on the clinical efficacy evaluation and reported adverse events, it can be concluded that the HTJWT is a safe and effective traditional chinese medicine for the treatment of patients suffering from chronic non-atrophic gastritis with mild to moderate symptoms.

**Clinical Trial Registration**: [www.clinicaltrials.gov], identifier [NCT04672018].

## 1 Introduction

In 2013, it was estimated that there were approximately 90 million new cases of gastritis, affecting roughly half of the global population. The prevalence of health problems due to gastritis is around 34.7% of the population in developed countries, whereas it affects about 50.8% of the population in developing countries (Global Burden of Disease Study, 2013 Collaborators, 2015; Wilkins et al., 2020; Feyisa and Woldeamanuel, 2021). Gastritis is caused by inflammation in the stomach lining. The inflammation of the gastric lining constituting two distinct presentations of the condition, namely, acute and chronic gastritis (Elseweidy, 2017). The condition is characterized by ongoing gastrointestinal discomfort in the stomach lining with two sub-types i.e., atrophic and non-atrophic (Sipponen and Maaroos, 2015; Rugge et al., 2020). The main difference between the two types of chronic gastritis is the extent of damage to the stomach lining. In comparison to atrophic gastritis, which causes more severe stomach lining inflammation, non-atrophic gastritis causes mild damage to the gastric linings (Schindler, 1966; Rugge et al., 2011). Depending on the type and degree of the condition, many symptoms may be present, but common ones include abdominal pain, bloating, nausea, vomiting, lack of appetite, and indigestion (Marcial et al., 2011). In more severe cases, vomiting, black stool from gastritis may signify internal bleeding. This inflammation may be caused by a variety of factors, including excessive alcohol use, routine use of nonsteroidal anti-inflammatory drugs (NSAIDs), and the infection caused by *H. pylori* (Weidner et al., 2009; Fang et al., 2018; Malfertheiner et al., 2023). The chronic non-atrophic gastritis (CNAG) is a type of gastritis referring to gastritis without atrophic changes in the gastric mucosa and infiltration of chronic inflammatory cells, mainly lymphocytes and plasma cells, in the gastric mucosa (Motoo et al., 1995; Rugge and Genta, 2005; Tang et al., 2012; Yan et al., 2019). It is a common disease of the digestive system with high prevalence rate and chronic nature. The lengthy treatment period often do not seriously affect the quality of life of patients (Rugge et al., 2002; Wang et al., 2019).

Herbal-based therapies are considered as an alternative option, especially by people who prefer natural remedies or worried about the possible adverse effects of conventional drugs (Tilburt and Kaptchuk, 2008; Chen et al., 2022). Additionally, when used properly, herbal medicines are generally regarded as safe and well-tolerated and have fewer adverse effects than the allopathic drugs (Fang et al., 2017; El-Dahiyat et al., 2020). The anti-inflammatory and antibacterial characteristics of some herbal-based drugs (Ghasemian et al., 2019), including HTJWT, is well documented. In addition, the HTJWT is a Chinese patent medicine (CN1368229A), it regulates Qi to relieve pain, abdominal distension, vomiting, and acid swallowing while controlling liver-stomach coordination (Tan et al., 2023). It also treats chronic gastritis, stomach, and duodenal ulcers, as well as conditions where the liver and stomach are not properly working (Dong-yun, 2012). The HTJWT is composed of six ingredients including *Hericium erinaceus mycelium, cuttlebone, Rhizoma corydalis* processed with vinegar, *Paeonia lactiflora* processed with alcohol, *Cyperus rotundus* processed with vinegar and *Glycyrrhiza*. After clinical verification, the total significant efficiency and total effective rate of the HTJWT in treating CNAG caused by disharmony between the liver and stomach were 63.95% and 89.83%, respectively. *Hericium mycelium* along with five TCMs are considered to be vitally important to treat CNAG caused by disharmony between liver and stomach (Liu et al., 1990; Wang et al., 2017; Mostoufi et al., 2018).

The HTJWT being a patent medicine (CN1368229A) has proven clinical efficacy in treating CNAG since 2001 with good safety profile and relatively no adverse reactions. Because of its application in the market and its main pharmacodynamic properties, the HTJWT could produce better effects on improving CNAG (Lu-qing et al., 2021). The purpose of this trial was to offer evidence that this HTJWT can be used as an alternative to conventional drugs for the treatment of CNAG. This trial was aimed to establish the non-inferiority effect of HTJWT compared to omeprazole tablet and also to clarify the clinical effectiveness and safety of it. The findings of this study will provide choices of alternate therapies and enhance patient outcomes for this widespread illness, which is essential for both clinicians and patients (Tan et al., 2023).

## 2 Methods and design

### 2.1 Study design

**FIGURE 1:**
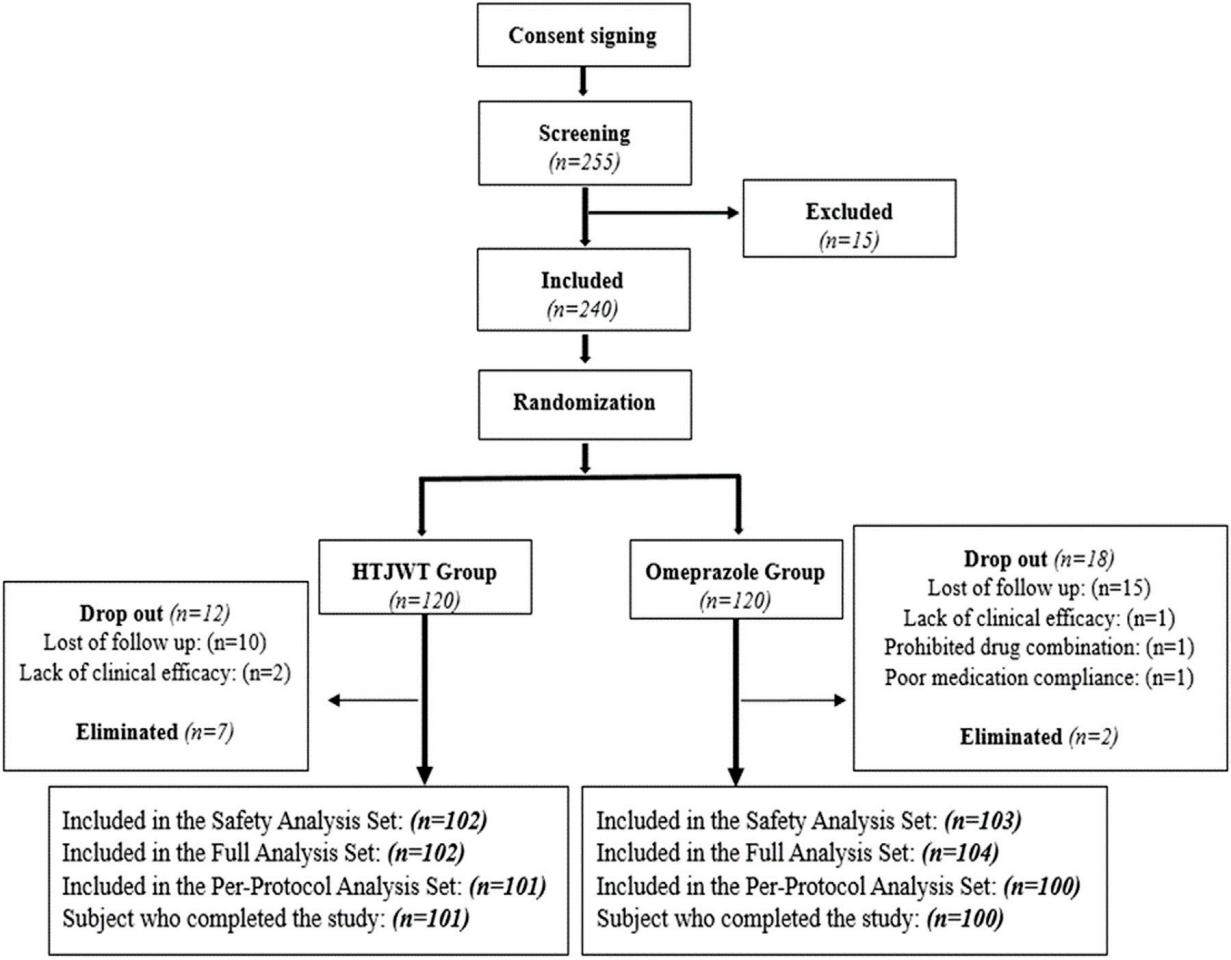
Shows the flowchart for subject screening, randomization and treatment.

This study is a phase II clinical trial that used randomization, double-blind and parallel-controlled methods to assess the efficacy and safety of the HTJWT in Pakistani individuals who had mild to moderate symptoms of CNAG. A total of 240 patients with CNAG diagnosed by gastroscopy and pathological biopsies were randomly divided into the HTJWT group and the Omeprazole group in a 1:1 ratio (Figure 1). The patients consumed the investigated drugs (i.e., treatment and controlled) up to 28 days. The primary efficacy indicators of the trial were to measure the improvement rate of main clinical symptoms (gastralgia and gastric distension) from day 0 to day 28. The study was carried out according to the ethical principles outlined in the Declaration of Helsinki, Good Clinical Practice Guidelines, and Drug Regulatory Authority of Pakistan. Additionally, the study adhered to all relevant legislation pertaining to new TCM drugs. After the approval from the Institutional Ethics Committee of the International Center for Chemical and Biological Sciences, (Ref.# ICCBS/CBSCR/IEC/LET-045/2021), the National Bioethics Committee of Pakistan, (Ref: No. 4-87/NBC-465) and the Drug Regulatory Authority of Pakistan, (License No. CT-0025), the study was executed. The trial was prospectively registered on www.clinicaltrials.gov with registration number: NCT04672018. All the patients provided written consent (informed consent form English/Urdu language) before participating in the trial-related activities.

### 2.2 Diagnostic criteria for CNAG

The diagnosis criteria for CNAG were based on the “Consensus Opinions on Chronic Gastritis in China” (Rugge et al., 2011), formulated by the Chinese Society of Gastroenterology and the Consensus Opinion on the Diagnosis and Treatment of Chronic Gastritis Combined of Integrated Traditional Chinese and Western Medicine (Fang et al., 2017) formulated by the Committee on Digestive System Diseases of the Chinese Association of Integrative Medicine (Ghasemian et al., 2019) and other relevant documents. The diagnosis of CNAG was based on the following criteria:

a. Clinical manifestation

Patients having non-specific dyspepsia, such as distention, epigastric pain, vomiting, acid regurgitation, nausea, belching, and loss of appetite were enrolled in the trial.

b. Physical indicators

Negligible changes were observed in case of most patients during the physical examinations however, only in few cases mild discomfort or soreness in the upper abdomen were observed while pressing the upper area of the stomach.

c. Evaluation by endoscopy and histopathology

The CNAG diagnosis was primarily relied on endoscopic and histopathological examinations. The typical endoscopy of patients suffering from non-atrophic gastritis exhibited the characteristics appearance of red plaques patches, punctuates and striae, uneven andcoarsal mucosa, hemorrhagic exudates and mucosal edematous. Whereas, the histopathological examination of the gastric mucosa showed no atrophic changes, dysplasia, intestinal metaplasia, or pseudo-pyloric gland metaplasia. In endoscopy the erythema, erosion, hemorrhage and bile reflux were evaluated by four grade, as shown in Supplementary Table S1, and the chronic and active inflammation were assessed with the help of histopathological examination as shown in Supplementary Table S2. As per the grading criteria selected for the evaluation of histopathological images, the patients with mild chronic inflammation is categorized into grade 1 while the patients with moderate chronic inflammation is categorized into grade II. The patients exhibited mild to moderate (i.e., grade I and grade II respectively) chronic inflammation were eligible to be included in the clinical trial.

### 2.3 Enrollment criteria

#### 2.3.1 Inclusion criteria

The inclusion criteria for the enrollment of the patients were; 1) who complied the diagnostic criteria of CNAG; 2) aged 18–65 years; and 3) voluntarily signed informed consent form to participate in the clinical trial.

#### 2.3.2 Exclusion criteria

The criteria for the exclusion of the patients were 1) a history of gastric surgery; 2) complicated and special types of gastritis, hemorrhage, peptic ulcer, cholelithiasis, cholecystitis, dysplasia of gastric mucosa or pathological diagnosis of suspected malignant change; 3) atrophy and/or intestinal metaplasia by pathological examination; 4) severe diseases associated with cardiovascular, cerebrovascular, kidney, liver, lung and hematopoietic system (above grade II of cardiac function; Creatinine value above the upper limit of normal), or serious diseases affecting their survival, such as acquired immunodeficiency syndrome (AIDS) or cancer; 5) to treat CNAG consumed chinese and/or western medicine in the last 2 weeks; 6) having psychiatric disorders or a history of taking alcohol or drug abuse; 7) pregnant and lactating women; 8) had a history of allergy from trial medication; 9) Those who have participated in other drug clinical trials during the last 3 months; 10) due to the undistinguished and complicated syndromes; 11) As per judgement of the investigator the subject participation in the clinical trial is inappropriate.

### 2.4 Sample size calculation

The data gather from clinicians prescribing HTJWT in China, the investigators believed that the overall effectiveness of HTJWT in the clinical treatment of CNAG would not be less than 60.00%. Therefore, the sample size calculation was based on the aforementioned observation and the required number of the patients were estimated accordingly. According to the calculation method for sample related to non-inferiority test in the second edition of Medical Statistical Methods (edited by Jin Pihuan, Fudan University Press, 2003,6), the sample size estimation method and sample size estimation formula for superiority clinical trials are as follows:

$$\text{Estimation formula}:\text{nc}=\left(Z1-\alpha +Z1-\beta \right)2\left[\pi C\left(1-\pi C\right)+\pi T\left(1-\pi T\right)\right]/\left(\Delta -\delta \right)2$$

Parameter setting:α=0.025,β=0.20

πC=0.86,πT=0.85

$$\delta =\pi C-\pi T=\left(0.86-0.85\right)=0.01$$

Δ=0.15

Calculation result: n ≈ 99 example

i.e., estimated by sample content in non-inferiority clinical trials using the formula α = 0.025. Under the set condition of 80% probability, the estimated minimum number of patients in the control group was 99. The 20% dropout rate was considered for the feasibility to conduct clinical trial with a sample size of 240 patients, that were equally divided into treatment and control groups, i.e., 120 patients in each.

### 2.5 Drug under investigation

The HTJWT; 0.38 g per tablet or placebo, manufactured by Hunan Xinhui Pharmaceutical Co., Ltd., China and the Omeprazole tablet; 20 mg per tablet or placebo, manufactured by Sinopharm Group Industry Co., Ltd. were used in this study. The HTJWT is composed of *Hericium Erinaceus Mycelium, cuttlebone, Rhizoma Corydalis* processed with vinegar, *Paeonia Lactiflora* processed with alcohol, *Cyperus Rotundus* processed with vinegar, and *Glycyrrhiza*. The blinding were maintained in terms of packaging, appearance and other characteristics of the HTJWT active and placebo to prevent any allocation bias. The investigational drugs were packaged according to the specified requirements of the Chinese Pharmacopoeia (Chinese Pharmacopoeia Commission, 2015).

### 2.6 Patients allocation and treatments

Subjects who met the criteria were split randomly into two groups. Patients in the treatment group received orally four HTJWT (0.38g/tablet), three times a day and one placebo of Omeprazole enteric-coated tablet prior to breakfast, daily. On the other hand, patients in the control group received one Omeprazole enteric-coated tablet (20 mg/tablet) prior to breakfast and four placebos of HTJWT, thrice a day. The patients consumed the investigated drugs (i.e., treatment and controlled) up to 28 days. The sponsor provided the allocation sequences, using a block randomization method generated by statistical analysis software (SAS, version 9.4) with 1:1 ratio between the treatment (HTJWT) and control (Omeprazole) drugs. This clinical trial was double-blinded, so the patients and investigators both were unaware about the allocation of intervention till the conclusion of the clinical trial. The HTJWT and Omeprazole drugs along with their simulants were packed in pre-coded similar boxes, according to the randomization sequence to assure the allocation concealment. The investigational drugs were stored as per the storage condition prescribed by the manufacturer and were dispensed to the patients according to the randomization. The data was analyzed by statisticians to assure that all enrolled patients were evenly assigned to the HTJWT or Omeprazole group. The principal investigator was authorized to carry out unblinding in case of serious adverse events (SAEs) or other undesirable occurrences in the clinical trial. The duration of the therapy was 28 days, and the subsequent appointment scheduled after the 28th day of medication administration was termed as the follow-up visit (Supplementary Table S3). Investigators conducted daily reviews of patients’ medication usage, wellbeing, and patient’s diary records via telephone calls during the whole trial period. The comprehensive evaluation timetable is specified in Supplementary Table S3.

### 2.7 Endpoints of study

The duration of treatment was up to 28 days and eligible patients were assessed through VAS and clinical symptoms on the 1st day (before 1st dose administration), 15 ± 2 days and after the 28 ± 5 days of the trial (Supplementary Table S4). The VAS consist of a 10 cm line with numeric pain intensity scale, (0 = painless and 10 cm = extreme pain). The patients were instructed to mark their level of gastric pain on the VAS accordingly. Clinical signs and symptoms of the patients were graded as shown in Supplementary Table S5, before 1^st^ dose, after 2 weeks and 4 weeks of investigational product administration. The efficacy and safety parameters of all patients were evaluated before the initiation of clinical trial and during follow-up period, specifically after the 28th day. The main safety parameters investigated for all patients included blood, urine, and stool routine tests, urine pregnancy test, ECG, liver function tests (alanine aminotransferase ALT, aspartate aminotransferase AST, total bilirubin T. BIL, γ-glutamate transpeptidase GGT, alkaline phosphatase), renal function tests (blood urea nitrogen BUN, creatinine Cr). The stool antigen test for detection of *H. pylori* were carried out in order to establish the end point of secondary efficacy.

#### 2.7.1 Efficacy endpoints

The primary efficacy endpoint of the study was; the improvement rate of main clinical symptoms and signs (*gastralgia and gastric distension*) as shown in Supplementary Table S5 obtained on day 1, day 15th and after 28th day treatment. The secondary efficacy endpoints were assessed through VAS for epigastric pain, improvement rate of secondary clinical symptoms and signs of CNAG, gastric pain and gastric distension disappearance/remission rate, gastric pain and gastric distension remission/disappearance time at day-1, day-15th and 28th day of drug administration. The eradication rate of *H. pylori* was carried out on day 28 for all patients. Clinical efficacy was determined by the combined scores of main symptoms (MS) and secondary symptoms (SS) then divided by MS to get efficacy index, including clinical remission, obvious effect, effective or ineffective. The improvement assessment of MS were gastralgia and gastric distension while abdominal pain, loss of appetite, bitterness and dryness in the mouth, lack of strength, nausea and vomiting, acid regurgitation, belching, upset and irritable were SS. Each patient’s score of MS and SS as well as total symptoms (TS) scores were calculated and compared with score of day 0 and follow-up for both groups. The recovery time from individual symptom, MS, SS, and TS scores at visits 1 to 4 is given in (Supplementary Table S4). The clinical efficacy of HTJWT was judged by the improvement of MS and SS scores, before and after treatment (Zhang et al., 2014; Chen et al., 2022).

The **efficacy index** as “yes”, was used to quantify the clinical efficacy. For **clinical recovery**: the clinical symptoms had essentially disappeared when the efficacy index was ≥95%; **Significantly effective**: significant improvement in clinical symptoms, with 70% ≤ efficacy index <95%; **Effective**: improvement in clinical symptoms, 30% ≤ effectiveness index <70%; and “No” for **Ineffective**: no clinical improvement in symptoms, with efficacy index below 30%. The following formula has been used to evaluate the clinical effectiveness of HTJWT after the completion of treatment;

$$Efficacy\;index\;\left(\%\right)=\frac{Pre-treatment\;integral-\;Post-treatment\;integral}{Pre-treatment\;integral}\times 100$$

Each patient was classified as being in clinical remission (efficacy index ≥95%), obvious effect (with 70% ≤ efficacy index <95%), effective (30% ≤ efficacy index <70%), or ineffective (efficacy index <30%) based on the examination of their effectiveness index. The investigators, who analyzed and evaluated the sign and symptoms of the patients remained blinded.

#### 2.7.2 Safety endpoints

A number of clinical investigations were carried out during the entire period of clinical trial which include measurement of blood pressure, pulse rate, body temperature, and respiratory rate. The ECG was recorded at screening and follow up visits. Blood, urine, and stool routine tests, as well as liver function tests (ALT, AST, TBIL, AKP, γ-GT), and renal function tests (BUN, Cr) on day 0 and on 28th ± 5 days were conducted for patients of both groups. Medication compliance and adverse events (AEs) were evaluated for all patients during the entire duration of trial (28th ± 5 days). All the AEs are recorded in detail that include remission, occurrence and severity in patient’s diary card which were transformed into case report form (CRF) for investigator’s review. The AEs are classified into mild (showing no symptoms or mild symptoms without requiring any treatment), moderate (requiring small, local, or non-invasive treatment), or severe (resulting in disability, hospital stay, or prolonged hospital stay; clinically significant but not instantly fatal).

### 2.8 Statistical analysis

The full analysis set (FAS) referred to all cases that were randomized and have consumed the study drug at least once along with post-medication evaluation data according to the intent to treat (ITT) principle. The cases lacking any follow-up data after enrollment (without any medication) were excluded. In this trial, FAS is the main population set for the analysis of efficacy data and the missing data related to efficacy is evaluated by last observation data carryover method (LOCF).

The per protocol set (PPS) referred to all cases that met the criteria for inclusion as per the protocol, like 80%–120% medication compliance, completed all follow up visits and the data related to indicators specified in the CRF. The cases included in the PPS analysis did not used treatments that may have affected the efficacy evaluation, and they did not violate the trial protocol. The PPS was finalized during blind data verification and was the secondary population for efficacy evaluation. The PPS data is used for the analysis of both primary and secondary efficacy outcomes.

The statistical analysis is carried out through the statistical analysis software (SAS, version 9.4). Descriptive statistical indicators for quantitative data include number of cases, mean, standard deviation, median, quartile, maximum, minimum, etc. The minimum and maximum values retained the same decimal places as the original data. The descriptive statistical indicators for qualitative data include the number of cases and frequency. Percentages are calculated to one decimal place using non-missing data. The rank sum test was used for inter-group comparison. The scores at visit 3 and visit 4 were compared before and after administration of investigational products, using a paired rank sum test. The main efficacy indicators were estimated using a non-inferiority test method, with a unilateral test level α = 0.025, with a non-inferiority margin of 0.15. The test drug non-inferiority is considered when the non-inferiority test *p*-value was <0.025 when compared with the control. A two-sided test with a test level of α = 0.05 is used for the statistical analysis of other indicators and the difference is deemed statistically significant at *p* < 0.05.

## 3 Results

### 3.1 Study completion

A total of 255 confirmed CNAG patients were identified and recruited from November 2022 to February 2023 as shown in Figure 1. Out of these patients, 15 were excluded from the trial, and the remaining eligible 240 patients were randomly divided into the treatment (HTJWT) group (*n* = 120) and the control (Omeprazole) group (*n* = 120) respectively in a ratio of 1:1 **(** Figure 1 **)**. A total of 19 patients from the treatment group and twenty subjects from the control group were excluded from further participation in the trial. Hence, the study concluded with a total of 201 patients who successfully participated and were considered for the final evaluation, specifically 101 subjects in the treatment group and 100 subjects in the control group. The patients recruited in the trial were belong to different ethnic groups. The mean age of patients in the treatment group was 37.3 ± 12.08 years, with 54.5% males and 45.5% females. There were 75.2% married and 24.8% unmarried; 49.5% manual workers and 50.5% professional workers. The average height, weight, and Body mass index (BMI) of the patients in the treatment group were 158.87 ± 10.070 cm, 74.22 ± 17.178 kg, and 29.32 ± 6.838 kg/m^2^ respectively. Similarly, the average age of patients in the control group was 37.4 ± 12.63 years, with 42.0% males and 58.0% females. There were 74.0% married and 26.0% unmarried patients in the control group and 34.0% of them were manual workers while 66.0% were professional workers. The average height, weight and BMI of the patients in the control group were 158.86 ± 9.799 cm, 71.04 ± 15.691 kg, and 27.99 ± 6.014 kg/m^2^ respectively (Table 1). Both groups exhibited negligible statistical differences in terms of age, gender, ethnicity, marital status, height, weight, BMI, etc. (*p* > 0.05) were observed between the groups however there was a statistically significant difference in terms of job nature (*p* < 0.05), with a larger proportion of professional workers in the control group.

**TABLE 1:** Demographic data and baseline characteristics of the subjects.

Variable		Treatment group	Control group	*p*-value
Age (year)	N	101	100	0.9213
	Mean (SD)	37.3 (12.08)	37.4 (12.63)	
	Min∼Max	18∼65	18∼65	
Gender	Male	55 (54.5%)	42 (42.0%)	0.0772
	Female	46 (45.5%)	58 (58.0%)	
	Total	101 (100.0%)	100 (100.0%)	
Ethnicity	Afghani	1 (1.0%)	1 (1.0%)	0.3807
	Balochi	4 (4.0%)	0 (0.0%)	
	Barmi	0 (0.0%)	1 (1.0%)	
	Chitrali	3 (3.0%)	1 (1.0%)	
	Gilgiti	1 (1.0%)	3 (3.0%)	
	Gujrati	1 (1.0%)	0 (0.0%)	
	Hindko	1 (1.0%)	0 (0.0%)	
	Kashmiri	0 (0.0%)	1 (1.0%)	
	Memon	2 (2.0%)	0 (0.0%)	
	Punjabi	14 (13.9%)	11 (11.0%)	
	Pushto	14 (13.9%)	17 (17.0%)	
	Sindhi	26 (25.7%)	30 (30.0%)	
	Urdu	34 (33.7%)	35 (35.0%)	
	Total	101 (100.0%)	100 (100.0%)	
Marital Status	Married	76 (75.2%)	74 (74.0%)	0.8390
	Unmarried	25 (24.8%)	26 (26.0%)	
	Total	101 (100.0%)	100 (100.0%)	
Profession	Manual Labor	50 (49.5%)	34 (34.0%)	0.0259
	Professional	51 (50.5%)	66 (66.0%)	
	Total	101 (100.0%)	100 (100.0%)	
Body Height (cm)	N	101	100	0.9936
	Mean (SD)	158.87 (10.070)	158.86 (9.799)	
	Min∼Max	136.0∼182.0	129.0∼179.0	
Body Weight (kg)	N	101	100	0.1724
	Mean (SD)	74.22 (17.178)	71.04 (15.691)	
	Min∼Max	32.0∼110.0	42.0∼110.0	
BMI (kg/m^2^)	N	101	100	0.1487
	Mean (SD)	29.31 (6.818)	27.99 (6.014)	
	Min∼Max	10.8∼47.7	13.2∼41.9	
History of smoking	Quit smoking	6 (5.9%)	4 (4.0%)	0.7764
	active smokers	4 (4.0%)	5 (5.0%)	
	Never smoked	91 (90.1%)	91 (91.0%)	
	Total	101 (100.0%)	100 (100.0%)	
amount of cigarette (per day)	N	4	5	0.2751
	Mean (SD)	3.8 (2.22)	2.2 (1.30)	
	Min∼Max	2∼7	1∼4	
Gastroscopy and pathological biopsy	non atrophic gastritis	101 (100.0%)	100 (100.0%)	
	Total	101 (100.0%)	100 (100.0%)	
*H. Pylori* Inspection	Negative	14 (13.9%)	22 (22.0%)	0.1324
	Positive	87 (86.1%)	78 (78.0%)	
	Total	101 (100.0%)	100 (100.0%)	

During the trial, a total of 15.8% of patients in the treatment group had concomitant medication, while 15.0% of patients in the control group had concomitant medication. No statistically significant difference were observed between the groups using the chi-square test (*p* = 0.8688). The PPS, analysis showed that the drug compliance of patients in both treatment and control groups was between 80% and 120%, with good drug compliance.

### 3.2 Comparison of symptom score and efficacy index

The efficacy index of the MS, SS, and TS score in the treatment and control group was calculated by grouping. Non-parametric rank sum test was used to compare the difference in efficacy index between the groups.

The FAS showed that 2.9% of cases were clinically cured in the treatment group. The drug was significantly effective in 59.8% cases, effective in 30.4% cases, and ineffective in 6.9% cases in the treatment group. On the other hand, 2.9% cases were clinically cured, and the drug was significantly effective in 44.7% cases, effective in 40.8% cases, and ineffective in 11.7% cases in the control group. The findings indicated a statistically significant difference in the efficacy index of the TS score (*p* = 0.0336), and the efficacy index of the treatment group was higher than that of the control group.

The PPS analysis showed that 3.0% cases were clinically cured in the treatment group. The treatment was significantly effective in 60.4% cases, effective in 30.7% cases, and ineffective in 5.9% cases. In the control group, 3.0% cases were clinically cured and the treatment was significantly effective in 46.0% cases, effective in 41.0% cases, and ineffective in 10.0% cases. A statistically significant difference in the efficacy index of the total symptom score (*p* = 0.0468) was observed hence the efficacy index of treatment group is higher than the control group (Supplementary Table S6).

### 3.3 Study outcomes

#### 3.3.1 Primary efficacy end points evaluation and comparison

The scores of MS (i.e., gastralgia and stomach distension) and the TS of each patient before the start and end of the study for both the treatmenttreatment and control groups were calculated and compared by using the FAS and PPS analysis. treatment The FAS showed that the clinical effective rate was 90.2% in the treatment group and 89.3% in the control group with no statistically significant difference between the two groups (*p* = 0.8362) as assessed through the Chi-square test. The difference in effective rates between the treatment group and the control group was −0.0742 to 0.0918 calculated with 95% CI. The lower limit of the 95% CI for the difference in effective rates among the groups was −0.0742, which was greater than −0.15 indicating the non-inferiority of the treatment group compared to the control group.

The PPS analysis revealed that the effective rate was 91.1% in the treatment group and 91.0% in control group. However, there was no statistically significant difference between the two groups (*p* = 0.9824) as determined by the Chi-square test. The 95% CI for calculating the difference in effective rates among the treated group and control group is −0.0781 to 0.0798. The lower limit of the 95% CI for the difference in effective rates between the groups was −0.0781, which is greater than −0.15, indicating non-inferiority of the treatment group in comparison with the control group (Supplementary Table S7).

#### 3.3.2 Secondary efficacy end points evaluation and comparison

Using the FAS and PPS analysis, each patient’s scores of SS (such as abdominal pain, loss of appetite, bitterness and dryness of the mouth, lack of strength, nausea and vomiting, acid regurgitation, belching, upset and irritability) as well as the TS of each patient in the treatment and control groups were calculated and compared with values obtained before start and end of the trial for both groups.

The **FAS** of secondary efficacy endpoints showed that the clinical effective rate was 93 (91.2%) in the treatment group and 87 (84.5%) in the control group with no statistically significant difference between the two groups (*p* = 0.1421) when assessed through Chi-square test. The 95% confidence limit for calculating the difference in effective rates among the treated group and control group was −0.0219 to 0.1561.

The **PPS** analysis showed that the effective rate was 93 (92.1%) in the treatment group and 87 (87.0%) in control group with no statistically significant difference between the two groups (*p* = 0.2392, 95% CI = −0.0336–0.1352) as shown in Supplementary Table S7.

a. Comparison of clinical efficacy and non-inferiority of total symptom score

The clinical effective rate according to the FAS was 91 (88.3%) in the control group and 95 (93.1%) in the treatment group with no statistically significant difference between the two groups (*p* = 0.2372: 95% CI = −0.0312–0.1269). The effective rate in the treatment group was 95 (94.1%), and in the control group was 90 (90.0%) in the PPS analysis with no statistically significant difference between the two groups (*p* = 0.2877; 95% CI = −0.0341–0.1153) as shown in Supplementary Table S7.

b. Non-inferiority comparison of the clinical effectiveness of VAS score

The assessment of the VAS score for individual secondary symptoms in the FAS showed 87 (86.1%) clinical effective rate in the treatment group and 77 (80.0%) in the control group with no statistically significant difference between the two groups (*p* = 0.1165) as shown in Supplementary Table S8. The 95% CI for calculating the difference in effective rates between the treatment and control groups was −0.0202 to 0.1896. The lower limit of the 95% CI for the difference in the effective rates between the treatment group and control group was greater than −0.15 (as shown in Supplementary Table S6), indicating the non-inferiority of the treatment group compared to the control group. The PPS analysis of 87 (87.0%) effective rate in the treatment group and 80 (80.0%) in the control group with negligible difference between the two groups (*p* = 0.1824). The 95% CI for calculating the difference in effective rates among the treatmentcontrol and treatment groups was −0.0324 to 0.1724 (Supplementary Table S6). The lower limit of the 95% CI for the difference in effective rates between the groups was −0.15 indicating non-inferiority of the treatment group to the control group.

c. Relief/disappearance rate and time of stomach pain symptoms

**FIGURE 2:**
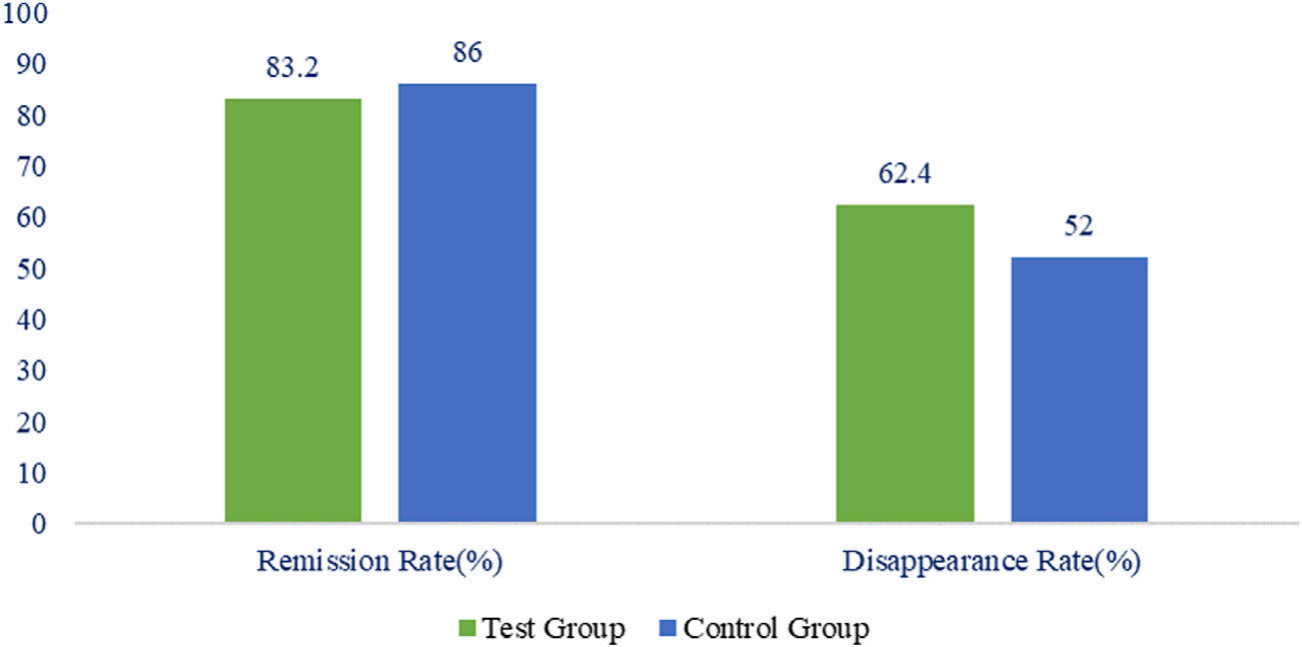
Percentage of remission and disappearance rate of stomach pain in the treatment and control group.

The remission rate of gastric pain symptoms in the treatment group was 84 (83.2%), while that in control group was 86 (86.0%) when assessed in the FAS. Negligible difference between the two groups (*p* = 0.5784) were observed as shown in Figure 2. The disappearance rate of gastric pain symptoms in treatment and control groups was 63 (62.4%) and 52 (52.0%) respectively with hardly any difference between the two groups (*p* = 0.1371) (Figure 2).

**FIGURE 3:**
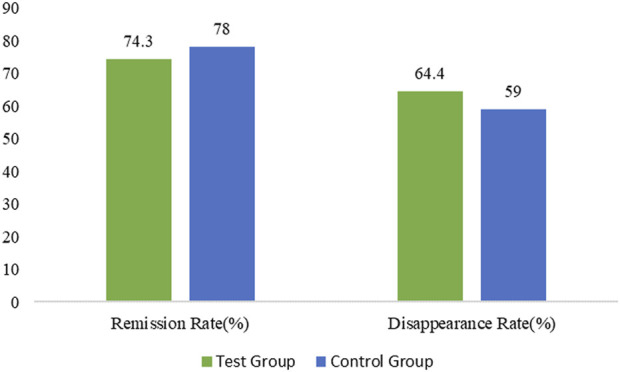
Percentage of gastric distension remission and disappearance rate in the treated and control group.

The remission time of gastric pain’s symptoms was 13.5 ± 5.15 days in the treatment group and 11.9 ± 4.69 days in the control group (Figure 3) with minor difference between the two groups (*p* = 0.0340). The relief time for gastric pain was comparatively shorter in the control group in comparison with the treatment group. The disappearance time of gastric pain’s symptoms in the treatment and control groups was 17.7 ± 3.33 and 16.8 ± 4.06 days respectively with no statistically significant difference between the two groups (*p* = 0.2632).

According to the PPS analysis, the remission rate of gastric pain’s symptoms in the treatment group and control groups was 84 (83.2%) and 86 (86.0%) correspondingly with no statistically significant difference between the two groups (*p* = 0.5784). The disappearance rate of gastric discomfort symptoms in treatment and control groups was 63 (62.4%) and 52 (52.0%) respectively with no statistically significant difference between the two groups (*p* = 0.1371).

The remission time for gastric pain symptoms in the treatment and control groups was 13.5 ± 5.15 and 11.9 ± 4.69 days respectively with no statistically significant difference between the two groups (*p* = 0.0430). The relief time for gastric pain in the control group was comparatively shorter than that in the treatment group. Similarly, the disappearance time of gastric pain’s symptoms in treatment and control groups was 17.7 ± 3.33 and 16.6 ± 4.15 days respectively with no statistically significant difference between the two groups (*p* = 0.1860) as showed in Supplementary Table S9.

d. Gastric distension relief/disappearance time

The remission rate of gastric distension symptoms in the treatment and control groups was 75 (74.3%) and 78 (78.0%) respectively with minor difference between the two groups (*p* = 0.5338) in the FAS. Similarly, the disappearance rate of gastric distension symptoms in the treatment and control groups was 65 (64.4%) and 59 (59.0%) respectively with no statistically significant difference between the two groups (*p* = 0.4348) in the FAS.

The remission time of gastric distension symptoms in the treatment and control groups was 11.2 ± 4.39 and 12.6 ± 4.99 days respectively with no statistically significant difference between the two groups (*p* = 0.0923). The disappearance time of gastric distension symptoms in the treatment and control groups was 13.8 ± 4.59 and 16.1 ± 4.64 days with no statistically significant difference among the two groups (*p* = 0.0116). The disappearance time of gastric distension symptoms in the treatment group was comparatively shorter in comparison with the control group.

In the PPS analysis, the remission rate of gastric distension symptoms in treatment and control groups was 75 (74.3%) and 78 (78.0%) respectively with no statistically significant difference among the two groups (*p* = 0.5338). The disappearance rate of gastric distension symptoms in the treatment and control groups was 65 (64.4%) and 59 (59.0%) respectively with no statistically significant difference among the two groups (*p* = 0.4348). The remission time for gastric distension symptoms in the treatment and control groups was 11.2 ± 4.39 and 12.6 ± 4.99 days respectively with no statistically significant difference between the two groups (*p* = 0.0923). The disappearance time for gastric distension symptoms in the treatment and control groups was 13.8 ± 4.59 and 16.1 ± 4.64 days respectively with a statistically significant difference among the two groups (*p* = 0.0116). The disappearance time for gastric distension symptoms in the treatment group was shorter than in the control group (Supplementary Table S9).

e. *Helicobacter pylori* negative rate/negative conversion rate

After medication of 4 weeks, the negative rate of *H. pylori* (assessed through stool antigen testing) was consistent in both the FAS and PPS analysis. The negative rate of *H. pylori* in the treatment and control groups was 70 (69.3%) and 76 (76.0%) respectively with no statistically significant difference between the two groups using the chi-square test (*p* = 0.2872) as shown in Table 2.

**TABLE 2:** *H. pylori* infection analysis and its negative conversion rate assessed through Stool Antigen Test [FAS, PPS].

Variable		Treatment group	Control group	*p*-value
Stool Antigen Test	Positive	31 (30.7%)	24 (24.0%)	0.2872
	Negative	70 (69.3%)	76 (76.0%)	
	Total	101 (100.0%)	100 (100.0%)	
*H. pylori* negative conversion rate				
		Treatment Group	Control Group	*p*-value
Converted into negative	No	26 (29.9%)	22 (28.2%)	0.8125
	Yes	61 (70.1%)	56 (71.8%)	
	Total	87 (100.0%)	78 (100.0%)	

The negative conversion rate of *H. pylori* was calculated as the proportion of patients with *H. pylori* that turned negative after treatment administration in both groups. The results of FAS and PPS were consistent. The negative conversion rate of *H. pylori* in the treatment and control groups was 61 (70.1%) and 56 (71.8%) with no statistically significant difference between the two groups using the Chi-square test (*p* = 0.8125) as shown in Table 2.

#### 3.3.3 Safety analysis

##### 3.3.3.1 Adverse events

The Safety analysis was carried out on **Safety Set** representing all cases randomized and used the study drug at least once with post-medication safety evaluation data. In this study, one subject reported 2 adverse events (epigastric pain and dyspepsia) in the control group and the incidence of adverse events was 0.5% (1/206) in the clinical trial. No incidence of adverse reactions was reported in this trial. The incidence of adverse events and adverse reactions in the treatment group was 0.0% (0/102). A total of 1 subject in the control group had 2 adverse events with an incidence of 1.0% (1/104) AEs and 0.0% (0/104) incidence of adverse reactions as shown in Table 3. No SAEs occurred in this trial indicating good safety of the administered treatments. There were no significant differences in respiratory rate, vital signs, systolic and diastolic blood pressure, laboratory reports for blood biochemistry, urine analysis, liver and renal function tests, and ECG evaluation between experimental and control group patients before and after treatment.

**TABLE 3:** Summary of the Adverse Events observed in the trial **[SS]**.

	Treatment group			Control group			Total		
	*N* = 102			*N* = 103			*N* = 205		
	Cases	*n*	Incidence (%)	Cases	*n*	Incidence (%)	Cases	*n*	Incidence (%)
Adverse events	0	0	0.0	2	1	1.0	2	1	0.5
Adverse drug reactions	0	0	0.0	0	0	0.0	0	0	0.0
Serious Adverse events	0	0	0.0	0	0	0.0	0	0	0.0

## 4 Discussion

Gastritis is the inflammation of outermost layer of the stomach lining, mostly caused by various factors mainly *H. pylori* infection, alcohol, and NSAIDs consumption for long-term (Annibale et al., 2002). In the theory of TCM, Chronic Gastritis (CG) can be categorized as stomachache, abdominal distention, or gastric discomfort, according to its clinical manifestations. As reported, CG is an underlying condition linked to various gastrointestinal and other organ illnesses that impair the quality of life and health of the affected individuals (Sipponen and Price, 2011). Thus, CG is regarded as an important public health concern by healthcare professionals and patients (Chen et al., 2022). Antibiotics, proton pump inhibitors (PPIs), gastro-protective agents, antacids, and gastro-prokinetics are the main medications frequently used for CNAG treatment (Fang et al., 2023). However, even with this standard therapy, the effectiveness is unsatisfactory and there are likely some unfavorable effects (den Hollander and Kuipers, 2012). According to Li’s research, long-term PPI use can change the mode of *H. pylori* colonization leading to an accelerated process of gland loss and subsequent development of chronic atrophic gastritis (Li et al., 2017). In addition, the rate of *H. pylori* eradication has fallen as a result of rising antimicrobial resistance and unsatisfactory patient medication compliance due to the side effects of the medications (Graham and Fischbach, 2010). In East Asia, the TCM treatment is frequently regarded as an alternate option for CG (Zhang et al., 2014). The TCM have recently been the research focus and attracted the attention of researchers in this context and thus may broaden the therapeutic and diagnostic options for CNAG (Li et al., 2013; Qin et al., 2013; Sun et al., 2013; Chen et al., 2022).

The HTJWT is composed of six ingredients including *H. mycelium*, *Cuttlebone*, *Vinegar Rhizoma Corydalis*, *Paeonia Lactiflora* (*Jiubaishao*), *Vinegar Xiangfu*, and *Glycyrrhiza*. All the ingredients of the HTJWT have definite therapeutic effects to regulates Qi, relieve abdominal pain, abdominal distension, vomiting, acid reflux, bloating and belching while controlling liver-stomach coordination (Tan et al., 2023) however, *H. mycelium* is the main ingredient of the formulation for treating CNAG (Liu et al., 1990; Ou-Yang, 2008; He and Dai, 2011; Rahnama et al., 2013; Thomas et al., 2015; Wang et al., 2017; Mostoufi et al., 2018). The purpose of this trial was to offer scientific evidence that this TCM can be used as an alternative for the treatment of CNAG. In Pakistan, the patients of CNAG are mainly treated through Omeprazole while in this trial the clinical efficacy and safety of Omperazole with HTJWT was compared which has not been done before. Furthermore, the clinical efficacy and safety of HTJWT was also never established on Patients of Pakistani ethnicity. The trial was concluded by an overall of 201 patients, with 101 patients in the treatment group and 100 patients in the control group. A total of 39 patients dropped out from the trial, comprising 19 patients from the treatment group and 20 patients from the control group, resulting in a dropout rate of 16.25%. The dropped-out patients in the treatment group consisted of those lost to follow-up (*n* = 10), lack of clinical efficacy (*n* = 2), and eliminated (*n* = 7) patients. Similarly, the dropped-out patients in the control group consisted of lost to follow-up (*n* = 15), lack of clinical efficacy (*n* = 1), concomitant medication (*n* = 1), and poor medication compliance (*n* = 1) patients.

During the trial, one patient in the control group experienced two mild to moderate AEs. Overall, the HTJWT was well tolerated and showed no clinically significant effects on routine blood tests, urinalysis, liver function tests, renal function tests, and the ECG findings. These findings provide evidence to support that HTJWT is a safe TCM for the treatment of patients suffering from mild to moderate CNAG. The clinical effective rate is 92 (91.1%) in the treatment group and 91 (91.0%) in the control group. Compared with Omeprazole (control), the clinical efficacy of HTJWT (treatment) is non-inferior along with a good safety profile. The clinical results showed that HTJWT is having curative effect on the remission of gastric distention, and the eradication of *H. pylori* (clearance rate 70.1%).

In this clinical trial a number of limitations and challenges were experienced, like; CNAG patients of Pakistani ethnicity were recruited in the clinical trial, which may restrict the geographical applicability of these findings. This study also excluded patients with severe comorbidities, who are more susceptible to the progression of stomach diseases. Further research is required to elucidate the molecular mechanisms through which HTJWT treats CNAG. We also hope that the findings of this study will spur additional investigation into how it works to treat the disease mechanistically. Further clinical trials should examine the effectiveness of longer treatment durations (i.e., 8–12 weeks), in preventing the recurrence of CNAG using a large sample size with a randomized, double-blind, and placebo-controlled trial design.

## 5 Conclusion

This investigation has confirmed the non-inferiority, efficacy, and safety of the HTJWT as a TCM in comparison with omeprazole. The treatment group patients had good medication compliance and excellent safety, particularly in terms of reducing gastric pain, gastric distension, and negative conversion rate of *H*. *pylori.* According to this clinical investigation, the HTJWT may offer a novel, safe alternative treatment for individuals with mild to severe CNAG.

## Data Availability

The original contributions presented in the study are included in the article/Supplementary Material, further inquiries can be directed to the corresponding author.
