# Supplementary material for: The safety and efficacy of Houtou Jianweiling tablet in patients with chronic non-atrophic gastritis: a double-blind, non-inferiority, randomized controlled trial
Source: Front Pharmacol. 2024 Feb 19;15:1293272. doi: 10.3389/fphar.2024.1293272 (PMC10911090; doi:10.3389/fphar.2024.1293272)
Supplement: Supplementary file 1 [file DataSheet1.docx]

**Supplementary Information File**

**The safety and efficacy of Houtou Jianweiling Tablet in patients with Chronic Non-atrophic Gastritis: a Double-Blind, Non-inferiority, Randomized Controlled Trial**

Muhammad Raza Shah*^1^, Samreen Fatima^1^, Sehrosh Naz Khan^1^, Shafiullah^1^, Zahid Azam^2^, Hafeezullah Shaikh^2^_,_ Shahid Majid^3^, He Chengdong^4^, Zhou Daijun^4^, Wei Wang^5^

^1^Center for Bioequivalence Studies and Clinical Research, Dr. Panjwani Center for Molecular Medicine and Drug Research, International Center for Chemical and Biological Sciences, University of Karachi, Karachi-75270.

^2^National Institute of Liver & GI Diseases (NILGID), DOW University of Health Sciences, Suparco Road, Gulzar-e-Hijri Scheme 33, Karachi, Sindh, Pakistan

^3^The Indus Hospital Karachi, Plot C-76, Sector 31/5, Opposite Korangi Crossing, Darussalam Society Sector 39, Karachi, Sindh, Pakistan

^4^Hunan Xinhui Pharmaceutical Co., Ltd., Wangcheng Economic and Technological Development Zone, Changsha City, Hunan Province, China

^5^TCM and Ethnomedicine Innovation & Development International Laboratory, School of Pharmacy, Hunan University of Chinese Medicine.

*Corresponding Author:

Professor Dr. Muhammad Raza Shah, Center for Bioequivalence Studies and Clinical Research, Dr. Panjwani Center for Molecular Medicine and Drug Research, International Center for Chemical and Biological Sciences, University of Karachi, Karachi-75270. Email: [raza.shah@iccs.edu](mailto:raza.shah@iccs.edu)

**Table S1: Endoscopic classification criteria for chronic gastritis**

| **Endoscopic characteristic** | **Endoscopic Features** | **Classification criteria** |
| --- | --- | --- |
| Erythema | Compared with the surrounding mucosa, it has obvious redness or flushing | Grade 0: None  GradeⅠ: Dispersed or discontinuous linear  Grade II: Dense spots or continuous lines  Grade III: Extensive integration |
| Erosion | Flat/protuberant verrucous: shallow mucosal lesions, flat or protuberant erosion of surrounding mucosa | Grade 0: None  Grade I: Single  Grade II: Multiple localities≤5  Grade III: Multiple and widespread≥6 |
| Hemorrhage | Intramucosal hemorrhage: punctate or flaky intramucosal hemorrhage with no prominent red or dark red bleeding spots (with/without bleeding, fresh/old) | Grade 0: None  Grade I: Local  Grade II: Multi-site  Grade III: Diffuse |
| Bile reflux | A lot of gastric juice, more green, and the pyloric is open. Bile flows from the duodenum to the stomach through the pylorus. | Grade 0: None  Grade I: Mucous are yellowish or yellowish green  Grade II: Between Grade Ⅰ and Grade III  Grade III: A large amount of yellow bile flows back into the stomach from the pylorus and yellow mucus adheres to the gastric mucosa. |

**Table S2: Histological variables grading and scoring criteria**

| **Histopathological changes** | **Grading standard** |
| --- | --- |
| Chronic inflammation | Grade 0: None |
|  | Grade Ⅰ: A few chronic inflammatory cells and limited to the superficial mucosa, not more than 1/3 of the mucosa. |
|  | Grade II: Chronic inflammatory cells are relatively dense and less than 2/3 of the mucosa. |
|  | +++: Chronic inflammatory cells are dense and occupy the whole mucosa. |
| Active inflammation | Grade 0: None |
|  | Grade Ⅰ: A few neutrophils infiltrated the lamina propria of the mucosa. |
|  | Grade Ⅱ: Neutrophils exists generally in the mucosa and can be seen on the surface of epithelial cells, pit epithelial cells, or glandular epithelial cells. |
|  | Grade Ⅲ: Neutrophils are dense or pit abscesses are seen in addition to moderate findings. |

**Table S3. Schedule of study assessments.**

| **Stage** | **Screening period** | | **Treatment Period** | | | **Follow-up**  **Period** |
| --- | --- | --- | --- | --- | --- | --- |
| **Visiting a doctor** | **Visit 1** | | **Visit 2** | | **Visit 3** | **Visit 4** |
| **Time** | **0 day** | | **Week 1**  **±2 days** | | **Weeks 3**  **±2 days** | **Weeks 5**  **±5 days** |
| **Collection of basic medical history** | | | | | | |
| Sign informed consent | √ | |  | |  |  |
| Fill in general information | √ | |  | |  |  |
| Past medical history and treatment history | √ | |  | |  |  |
| Complication with diseases and symptoms | √ | |  | |  |  |
| Physical examination | √ | |  | |  | √ |
| Combined medication | √ | | √ | | √ | √ |
| Criteria for admission | √ | |  | |  |  |
| **Diagnostic testing** | | | | | | |
| Gastroscopy and pathological biopsy | √ | |  |  | |  |
| **Exclusive testing** | | | | | | |
| Transabdominal ultrasound | √ | |  |  | |  |
| **Efficacy observation and diagnosis** | | | | | | |
| Main Symptom Score | | √ | √ | √ | | √ |
| Secondary Symptom Score | | √ | √ | √ | | √ |
| VAS score | | √ | √ | √ | | √ |
| **Safety Testing** | | | | | | |
| Vital signs | | √ | √ | √ | | √ |
| Routine blood test | | √ |  |  | | √ |
| Urine routine test | | √ |  |  | | √ |
| Stool routine test | | √ |  |  | | √ |
| Stool Antigen Test | |  |  |  | | √ |
| Liver and renal function | | √ |  |  | | √ |
| Electrocardiogram | | √ |  |  | | √ |
| Urine pregnancy | | √ |  |  | | √ |
| **Observation of adverse events** | | | | | | |
| Adverse Event Record | |  | √ | √ | | √ |
| **Other work** | | | | | | |
| Random grouping | |  | √ |  | |  |
| Dispensing drugs | |  | √ | √ | |  |
| Quantity, recovery and statistics of the drugs | |  |  | √ | | √ |

**Table S4. Clinical signs and symptoms**

|  | | | **Visit 1** | | | **Visit 2** | | | **Visit 3** | | | **Visit 4** | |
| --- | --- | --- | --- | --- | --- | --- | --- | --- | --- | --- | --- | --- | --- |
| **Variable** | **Result** | **HTJWT** | | **Control** | **HTJWT** | | **Control** | **HTJWT** | | **Control** | **HTJWT** | | **Control** |
| Gastralgia(Degree) | N | 101 | | 100 | 101 | | 100 | 101 | | 100 | 101 | | 100 |
|  | Mean(SD) | 3.0(1.08) | | 3.3(1.24) | 3.0(1.08) | | 3.3(1.24) | 2.0(0.85) | | 2.1(1.06) | 0.8(1.10) | | 1.1(1.25) |
|  | Min~Max | 2~6 | | 2~6 | 2~6 | | 2~6 | 0~4 | | 0~4 | 0~4 | | 0~4 |
|  |  |  | |  |  | |  |  | |  |  | |  |
| Gastralgia(Duration) | N | 101 | | 100 | 101 | | 100 | 101 | | 100 | 101 | | 100 |
|  | Mean(SD) | 3.0(1.15) | | 3.3(1.28) | 3.0(1.15) | | 3.3(1.28) | 2.0(0.87) | | 2.1(1.04) | 0.8(1.11) | | 1.1(1.25) |
|  | Min~Max | 2~6 | | 2~6 | 2~6 | | 2~6 | 0~4 | | 0~4 | 0~4 | | 0~4 |
|  |  |  | |  |  | |  |  | |  |  | |  |
| Gastralgia(Frequency) | N | 101 | | 100 | 101 | | 100 | 101 | | 100 | 101 | | 100 |
|  | Mean(SD) | 2.9(1.09) | | 3.2(1.19) | 2.9(1.09) | | 3.2(1.19) | 2.0(0.85) | | 2.0(0.99) | 0.8(1.10) | | 1.1(1.22) |
|  | Min~Max | 1~6 | | 1~6 | 1~6 | | 1~6 | 0~4 | | 0~4 | 0~4 | | 0~4 |
|  |  |  | |  |  | |  |  | |  |  | |  |
| Gastric Distension(Degree) | N | 101 | | 100 | 101 | | 100 | 101 | | 100 | 101 | | 100 |
|  | Mean(SD) | 2.4(1.30) | | 2.7(1.37) | 2.4(1.30) | | 2.7(1.37) | 1.1(1.15) | | 1.6(1.21) | 0.5(0.96) | | 0.7(1.11) |
|  | Min~Max | 0~4 | | 0~6 | 0~4 | | 0~6 | 0~4 | | 0~4 | 0~4 | | 0~4 |
|  |  |  | |  |  | |  |  | |  |  | |  |
| Gastric Distension(Duration) | N | 101 | | 100 | 101 | | 100 | 101 | | 100 | 101 | | 100 |
|  | Mean(SD) | 2.4(1.37) | | 2.7(1.46) | 2.4(1.37) | | 2.7(1.46) | 1.1(1.15) | | 1.6(1.18) | 0.5(0.96) | | 0.7(1.07) |
|  | Min~Max | 0~6 | | 0~6 | 0~6 | | 0~6 | 0~4 | | 0~4 | 0~4 | | 0~4 |
|  |  |  | |  |  | |  |  | |  |  | |  |
| Gastric Distension(Frequency) | N | 101 | | 100 | 101 | | 100 | 101 | | 100 | 101 | | 100 |
|  | Mean(SD) | 2.3(1.26) | | 2.6(1.35) | 2.3(1.26) | | 2.6(1.35) | 1.1(1.18) | | 1.5(1.13) | 0.5(0.98) | | 0.6(1.02) |
|  | Min~Max | 0~5 | | 0~6 | 0~5 | | 0~6 | 0~4 | | 0~4 | 0~4 | | 0~4 |
|  |  |  | |  |  | |  |  | |  |  | |  |
| **Total score of main symptoms** | N | 101 | | 100 | 101 | | 100 | 101 | | 100 | 101 | | 100 |
|  | Mean(SD) | 15.9(5.81) | | 17.8(6.70) | 15.9(5.81) | | 17.8(6.70) | 9.2(4.71) | | 10.8(5.51) | 3.9(5.21) | | 5.3(5.96) |
|  | Min~Max | 6~30 | | 6~36 | 6~30 | | 6~36 | 0~24 | | 0~24 | 0~24 | | 0~24 |
|  |  |  | |  |  | |  |  | |  |  | |  |
| Abdominal Pain | N | 101 | | 100 | 101 | | 100 | 101 | | 100 | 101 | | 100 |
|  | Mean(SD) | 2.0(0.00) | | 2.0(0.00) | 2.0(0.00) | | 2.0(0.00) | 1.8(0.57) | | 1.8(0.65) | 0.8(0.98) | | 1.0(1.00) |
|  | Min~Max | 2~2 | | 2~2 | 2~2 | | 2~2 | 0~2 | | 0~2 | 0~2 | | 0~2 |
|  |  |  | |  |  | |  |  | |  |  | |  |
| Loss of appetite | N | 101 | | 100 | 101 | | 100 | 101 | | 100 | 101 | | 100 |
|  | Mean(SD) | 1.1(0.92) | | 1.1(0.78) | 1.1(0.92) | | 1.1(0.78) | 0.8(0.82) | | 0.8(0.72) | 0.7(0.74) | | 0.7(0.74) |
|  | Min~Max | 0~3 | | 0~3 | 0~3 | | 0~3 | 0~3 | | 0~3 | 0~3 | | 0~3 |
|  |  |  | |  |  | |  |  | |  |  | |  |
|  |  |  | |  |  | |  |  | |  |  | |  |
| Bitterness and dryness in the mouth | N | 101 | | 100 | 101 | | 100 | 101 | | 100 | 101 | | 100 |
|  | Mean(SD) | 1.8(1.17) | | 1.9(1.21) | 1.8(1.17) | | 1.9(1.21) | 1.0(0.85) | | 1.0(0.84) | 0.9(0.70) | | 0.8(0.70) |
|  | Min~Max | 0~3 | | 0~3 | 0~3 | | 0~3 | 0~3 | | 0~3 | 0~3 | | 0~3 |
|  |  |  | |  |  | |  |  | |  |  | |  |
| Lack of strength | N | 101 | | 100 | 101 | | 100 | 101 | | 100 | 101 | | 100 |
|  | Mean(SD) | 1.2(0.55) | | 1.1(0.46) | 1.2(0.55) | | 1.1(0.46) | 1.0(0.53) | | 1.0(0.52) | 0.9(0.49) | | 0.9(0.49) |
|  | Min~Max | 0~3 | | 0~2 | 0~3 | | 0~2 | 0~3 | | 0~3 | 0~2 | | 0~2 |
|  |  |  | |  |  | |  |  | |  |  | |  |
| Nausea and vomiting | N | 101 | | 100 | 101 | | 100 | 101 | | 100 | 101 | | 100 |
|  | Mean(SD) | 1.1(1.00) | | 1.1(1.00) | 1.1(1.00) | | 1.1(1.00) | 0.2(0.57) | | 0.2(0.60) | 0.1(0.40) | | 0.0(0.28) |
|  | Min~Max | 0~2 | | 0~2 | 0~2 | | 0~2 | 0~2 | | 0~2 | 0~2 | | 0~2 |
|  |  |  | |  |  | |  |  | |  |  | |  |
| Acid regurgitation | N | 101 | | 100 | 101 | | 100 | 101 | | 100 | 101 | | 100 |
|  | Mean(SD) | 1.6(0.83) | | 1.5(0.86) | 1.6(0.83) | | 1.5(0.86) | 0.6(0.93) | | 0.8(0.98) | 0.2(0.55) | | 0.4(0.79) |
|  | Min~Max | 0~2 | | 0~2 | 0~2 | | 0~2 | 0~2 | | 0~2 | 0~2 | | 0~2 |
|  |  |  | |  |  | |  |  | |  |  | |  |
| Belching (irritable) | N | 101 | | 100 | 101 | | 100 | 101 | | 100 | 101 | | 100 |
|  | Mean(SD) | 0.9(0.99) | | 1.0(1.00) | 0.9(0.99) | | 1.0(1.00) | 0.2(0.54) | | 0.3(0.74) | 0.1(0.34) | | 0.1(0.48) |
|  | Min~Max | 0~2 | | 0~2 | 0~2 | | 0~2 | 0~2 | | 0~2 | 0~2 | | 0~2 |
|  |  |  | |  |  | |  |  | |  |  | |  |
| Upset and irritable | N | 101 | | 100 | 101 | | 100 | 101 | | 100 | 101 | | 100 |
|  | Mean(SD) | 1.7(0.73) | | 1.6(0.77) | 1.7(0.73) | | 1.6(0.77) | 1.4(0.90) | | 1.4(0.90) | 1.2(0.98) | | 1.3(0.95) |
|  | Min~Max | 0~2 | | 0~2 | 0~2 | | 0~2 | 0~2 | | 0~2 | 0~2 | | 0~2 |
|  |  |  | |  |  | |  |  | |  |  | |  |
| Total score of secondary symptoms | N | 101 | | 100 | 101 | | 100 | 101 | | 100 | 101 | | 100 |
|  | Mean(SD) | 11.2(2.88) | | 11.2(2.67) | 11.2(2.88) | | 11.2(2.67) | 7.1(2.52) | | 7.3(2.38) | 4.8(2.07) | | 5.2(2.36) |
|  | Min~Max | 5~18 | | 4~17 | 5~18 | | 4~17 | 0~16 | | 2~17 | 0~12 | | 0~14 |
|  |  |  | |  |  | |  |  | |  |  | |  |
| **Total syndrome score** | N | 101 | | 100 | 101 | | 100 | 101 | | 100 | 101 | | 100 |
|  | Mean(SD) | 27.1(7.03) | | 29.1(7.90) | 27.1(7.03) | | 29.1(7.90) | 16.3(6.23) | | 18.1(6.55) | 8.7(6.52) | | 10.5(7.43) |
|  | Min~Max | 16~47 | | 10~47 | 16~47 | | 10~47 | 0~37 | | 2~36 | 0~33 | | 0~31 |

**Table S5. Grading and scoring criteria of main symptoms**

| **Main symptoms** | | **Scoring Standard** | **Score** |
| --- | --- | --- | --- |
| **Gastralgia** | Degree | **0**: None before treatment or disappeared after treatment | **0** |
|  |  | **2 points**: Slight stomachache, no impact on work and rest, VAS score 1-3 | **2** |
|  |  | **4 points**: Stomach pain can tolerable, attack frequently, affect work and rest, VAS score 4-6 | **4** |
|  |  | **6 points**: The stomach pain is intolerable and persist for a long time. Often needs pain killer to relief pain, VAS score is 7-10 | **6** |
|  | Duration | **0**: none before treatment or disappeared after treatment | **0** |
|  |  | **2 points**: Less than 1 hour a day | **2** |
|  |  | **4 points**: 1-2 hours a day | **4** |
|  |  | **6 points**: More than 2 hours a day | **6** |
|  | Frequency | **Points** = The number of days with symptoms per week, such as 7 points per day. |  |
| **Gastric distension** | Degree | **0**: None before treatment or disappeared after treatment | **0** |
|  |  | **2 points**: Slight gastric distension, from time to time, does not affect work and rest. | **2** |
|  |  | **4 points**: Gastric distension can tolerable, attacks frequently, affecting work and rest. | **4** |
|  |  | **6 points**: The gastric distension is intolerable and persist for a long time. Often needs pain killer to relief pain. | **6** |
|  | Duration | **2 points**: daily pain within 1 hour | **2** |
|  |  | **4 points**: daily pain within 1-2 hours | **4** |
|  |  | **6 points**: pain more than 2 hours a day | **6** |
|  | Frequency | **Points** = number of symptomatic days per week, 7 points for daily attack |  |
| **Total score of main symptoms** | | |  |

**Table S6: Symptom score effective index and clinical significance comparison between treatment group and control group**

| **Variable** | **Datasets** | **clinical significance** | **HTJWT Grp** | **Control Grp** | **P value** |
| --- | --- | --- | --- | --- | --- |
| Total score of main symptoms | FAS | Clinical remission | 54(52.9%) | 44(42.7%) | 0.2054 |
|  |  | Obvious effect | 10(9.8%) | 13(12.6%) |  |
|  |  | Effective | 28(27.5%) | 35(34.0%) |  |
|  |  | Ineffective | 10(9.8%) | 11(10.7%) |  |
|  |  | Total | 102(100.0%) | 103(100.0%) |  |
|  |  |  |  |  |  |
| Total score of secondary symptoms | FAS | Clinical remission | 2(2.0%) | 2(1.9%) | 0.6510 |
|  |  | Obvious effect | 17(16.7%) | 20(19.4%) |  |
|  |  | Effective | 74(72.5%) | 65(63.1%) |  |
|  |  | Ineffective | 9(8.8%) | 16(15.5%) |  |
|  |  | Total | 102(100.0%) | 103(100.0%) |  |
|  |  |  |  |  |  |
| Total syndrome score | FAS | Clinical remission | 3(2.9%) | 3(2.9%) | 0.0336 |
|  |  | Obvious effect | 61(59.8%) | 46(44.7%) |  |
|  |  | Effective | 31(30.4%) | 42(40.8%) |  |
|  |  | Ineffective | 7(6.9%) | 12(11.7%) |  |
|  |  | Total | 102(100.0%) | 103(100.0%) |  |
|  |  |  |  |  |  |
| Total score of main symptoms | PPS | Clinical remission | 54(53.5%) | 44(44.0%) | 0.2643 |
|  |  | Obvious effect | 10(9.9%) | 13(13.0%) |  |
|  |  | Effective | 28(27.7%) | 34(34.0%) |  |
|  |  | Ineffective | 9(8.9%) | 9(9.0%) |  |
|  |  | Total | 101(100.0%) | 100(100.0%) |  |
|  |  |  |  |  |  |
| Total score of secondary symptoms | PPS | Clinical remission | 2(2.0%) | 2(2.0%) | 0.8522 |
|  |  | Obvious effect | 17(16.8%) | 20(20.0%) |  |
|  |  | Effective | 74(73.3%) | 65(65.0%) |  |
|  |  | Ineffective | 8(7.9%) | 13(13.0%) |  |
|  |  | Total | 101(100.0%) | 100(100.0%) |  |
|  |  |  |  |  |  |
| Total syndrome score | PPS | Clinical remission | 3(3.0%) | 3(3.0%) | 0.0468 |
|  |  | Obvious effect | 61(60.4%) | 46(46.0%) |  |
|  |  | Effective | 31(30.7%) | 41(41.0%) |  |
|  |  | Ineffective | 6(5.9%) | 10(10.0%) |  |
|  |  | Total | 101(100.0%) | 100(100.0%) |  |

**Table S7: Symptom score effective index comparison between treatment group and control group**

|  |  | **Difference test** | | | **Non-inferiority test** | | **95 %CI** | |
| --- | --- | --- | --- | --- | --- | --- | --- | --- |
| **Variable** | **Datasets** | **HTJWT Grp** | **Control Grp** | **P value** | **P value** | **Lower** | | **Upper** |
| Total score of main symptoms | FAS | 92(90.2%) | 92(89.3%) | 0.8362 | <.0001 | -0.0742 | | 0.0918 |
| Total score of main symptoms | PPS | 92(91.1%) | 91(91.0%) | 0.9824 | <.0001 | -0.0781 | | 0.0798 |
| Total score of secondary symptoms | FAS | 93(91.2%) | 87(84.5%) | 0.1421 | <.0001 | -0.0219 | | 0.1561 |
| Total score of secondary symptoms | PPS | 93(92.1%) | 87(87.0%) | 0.2392 | <.0001 | -0.0336 | | 0.1352 |
| Total syndrome score | FAS | 95(93.1%) | 91(88.3%) | 0.2372 | <.0001 | -0.0312 | | 0.1269 |
| Total syndrome score | PPS | 95(94.1%) | 90(90.0%) | 0.2877 | <.0001 | -0.0341 | | 0.1153 |

**Table S8: VAS score effective index comparison between treatment group and control group**

|  | | | **Difference test** | **Non-inferiority test** | **95 %CI** | |
| --- | --- | --- | --- | --- | --- | --- |
| **Datasets** | **HTJWT Group** | **Control Group** | **P value** | **P value** | **Lower** | **Upper** |
| FAS | 87(86.1%) | 80(77.7%) | 0.1165 | <.0001 | -0.0202 | 0.1896 |
| PPS | 87(87.0%) | 80(80.0%) | 0.1824 | <.0001 | -0.0324 | 0.1724 |

**Table S9: Determination of gastralgia/gastric distention disappearance [PPS]**

| **Variable** |  | **HTJWT Grp** | **Control Grp** | **P value** |
| --- | --- | --- | --- | --- |
| Relief of gastralgia symptoms? | No | 17(16.8%) | 14(14.0%) | 0.5784 |
|  | Yes | 84(83.2%) | 86(86.0%) |  |
|  | Total | 101(100.0%) | 100(100.0%) |  |
|  |  |  |  |  |
| relief of gastric distention? | No | 26(25.7%) | 22(22.0%) | 0.5338 |
|  | Yes | 75(74.3%) | 78(78.0%) |  |
|  | Total | 101(100.0%) | 100(100.0%) |  |
|  |  |  |  |  |
| disappearance of gastralgia symptoms? | No | 38(37.6%) | 48(48.0%) | 0.1371 |
|  | Yes | 63(62.4%) | 52(52.0%) |  |
|  | Total | 101(100.0%) | 100(100.0%) |  |
|  |  |  |  |  |
| disappearance of gastric distention? | No | 36(35.6%) | 41(41.0%) | 0.4348 |
|  | Yes | 65(64.4%) | 59(59.0%) |  |
|  | Total | 101(100.0%) | 100(100.0%) |  |
|  |  |  |  |  |
| Relief time of gastralgia symptoms | N | 84 | 86 | 0.0340 |
|  | Mean(SD) | 13.5(5.15) | 11.9(4.69) |  |
|  | Min ~ Max | 3~26 | 3~23 |  |
|  |  |  |  |  |
| Relief time of gastric distention | N | 75 | 78 | 0.0923 |
|  | Mean(SD) | 11.2(4.39) | 12.6(4.99) |  |
|  | Min ~ Max | 3~23 | 2~23 |  |
|  |  |  |  |  |
| disappearance Time of gastralgia symptoms | N | 63 | 52 | 0.1860 |
|  | Mean(SD) | 17.7(3.33) | 16.6(4.15) |  |
|  | Min ~ Max | 6~25 | 7~23 |  |
|  |  |  |  |  |
| disappearance Time of gastric distention | N | 65 | 59 | 0.0116 |
|  | Mean(SD) | 13.8(4.59) | 16.1(4.64) |  |
|  | Min ~ Max | 5~23 | 6~26 |  |

**Investigation data of Houtou Jianweiling tablet stability**

**Drug name:** Houtou Jianweiling Tablet

**Packing**：0. 38g per tablet

**Product Performance Overview：**

The main ingredients of this product are Extract of Hericium mycelium, Cuttlebone, Vinegar Rhizoma Corydalis, Paeonia Lactiflora (Jiubaishao), Vinegar Xiangfu, and Glycyrrhiza. The tablet contains the brownish-yellow powder with aromatic sweet smell and slightly bitter taste. Smoothing the liver and regulating the stomach. It is indicated for treating incoordination between the liver and the stomach, distension and pain of the epigastric and coastal regions, vomiting and acid regurgitation, gastritis and duodenal ulcer with symptoms described above.

Product implementation standards, inspection items and inspection batches:

| **Executive standard:** YBZ08172009  《Chinese Pharmacopoeia》2015 II and IV Implemented on December 01, 2015 |
| --- |
| Inspection items: traits, identification, weight difference, disintegration time limit, content determination, microbial limit. |
| Inspection batches: A total of 7 batches were inspected:  2016：160301  2017：170101、170201、170501、170701  2018：180101  2019：190101 |

**Validity period:** The validity period of this product is 18 months, and the inspection period is 24 months.

**Product inspection:** Each batch was sampled and tested according to the inspection cycle, frequency, and inspection items. No abnormalities occurred. The results of the 17-year inspection batch are as follows:

| Houtou Jianweiling Tablet Stability Investigation - Disintegration Time Limit, Content (mg) Test Results | | | | | | | | |
| --- | --- | --- | --- | --- | --- | --- | --- | --- |
| Batch number | Project | 0months | 3months | 6months | 9months | 12months | 18months | 24months |
| 170201 | Disintegration Time Limit | 18 | 30 | 22 | 21 | 14 | 25 | 26 |
|  | content(mg/tablet) | 0.78 | 0.71 | 0.77 | 0.79 | 0.86 | 0.77 | 0.77 |
| 170501 | Disintegration Time Limit | 15 | 20 | 23 | 23 | 22 | 13 | 33 |
|  | content(mg/tablet) | 0.76 | 0.73 | 0.75 | 0.78 | 0.72 | 0.71 | 0.74 |
| 170701 | Disintegration Time Limit | 22 | 27 | 20 | 17 | 24 | 25 | 29 |
|  | content(mg/tablet) | 0.78 | 0.91 | 0.83 | 0.86 | 0.87 | 0.95 | 0.85 |

**Conclusion:** The results show that the disintegration term has a rising trend, but the term of validity conforms to the quality standard. Other terms are in conformity with the provisions and there is no significant change.
